# Everyday experiences of post-diagnosis life with dementia: A co-produced photography study

**DOI:** 10.1177/1471301220973632

**Published:** 2020-11-26

**Authors:** Jemima Dooley, Joe Webb, Roy James, Harry Davis, Sandy Read

**Affiliations:** Centre for Academic Primary Care, Bristol Medical School, 1980 University of Bristol, UK; Norah Fry Centre for Disability Studies, School for Policy Studies, 1980 University of Bristol, UK; Member of the Forget-Me-Not Research Group, UK; Member of the Forget-Me-Not Research Group, UK; Member of the Forget-Me-Not Research Group, UK

**Keywords:** post-diagnosis, dementia, photography, qualitative research, coproduction

## Abstract

There has been surprisingly little research capturing people’s everyday lives in the early years following a diagnosis of dementia. This project was co-produced by three people with dementia and two university researchers. The co-researchers with dementia formulated the aims of this project as: (1) to explore post-diagnosis life with dementia and (2) to use data collection methods as a form of peer support and confidence building for the participants. The intent was to provide the opportunity to learn new skills and support participants to share their experiences without putting them on the spot. Five participants with recent diagnoses received a photography lesson and cameras to take photographs of their everyday lives. This was followed with a focus group in which the photographs were discussed. The participants used their photographs to explain: (1) the differences between their past and present with dementia, (2) how dementia affected their thought processes, (3) pets and grandchildren as facilitators of reciprocal joy and support, (4) the emotional effects of living with a dementia diagnosis, and (5) the solace and stability of nature in a changing world. The participants’ creative use of photography supported them to express the complex emotions felt after a diagnosis of dementia and they reported the benefit of doing this in an environment with peers going through the same experiences. The role of the co-researchers with dementia was the key to the success of this project, drawing on their own experiences to design the project and support the participants. Future research should draw on the experiences of people with dementia to design research projects and care interventions.

## Introduction

Receiving a diagnosis of dementia is a life-changing event that can lead to feelings of loss, denial, anger and a negative impact on individuals’ identity and self-worth (Aminzadeh et al., 2007; Bunn et al., 2012; Clare et al., 2008; Xanthopoulou & McCabe, 2019). Much of the rhetoric surrounding dementia diagnosis disclosure focuses on ‘living well’ and planning for the future (Carpenter & Dave, 2004; Martyr et al., 2018). However, the lack of support for people with dementia post-diagnosis has been well-documented (Hagan, 2020; Innes et al., 2014). As a result, people in the early stages of dementia report developing their own coping styles and support systems (Droes et al., 2011; Gellert et al., 2018; Lamont et al., 2019).

Research into receiving a dementia diagnosis has focused on the delivery itself (Dooley et al., 2015; Low et al., 2018; Poyser & Tickle, 2019), or the diagnosis as a ‘journey’ from symptom recognition to immediate reactions (Campbell et al., 2016; Robinson et al., 2011; Xanthopoulou & McCabe, 2019). There has been markedly less focus on capturing the lived experience of ‘what happens next’: the first years post diagnosis where people learn to live successfully, or often struggle, with the changes they are experiencing (Mason et al., 2005; Brooker et al., 2017; Bartlett et al., 2017; Vernooij-Dassen et al., 2006). Furthermore, there is a focus on caregivers (Monteiro et al., 2018), or joint conversations with spouses (Gellert et al., 2018), and not the person with dementia (Aminzadeh et al., 2007). This is despite a growing acknowledgement of the rights of people living with dementia to have their experiences included and be active participants in research (Hubbard et al., 2003; McKeown et al., 2010; Shakespeare et al., 2019; Williams et al., 2020).

The cognitive symptoms of dementia, including memory loss and word finding difficulties, can cause methodological challenges to research (Carmody et al., 2015; Webb et al., 2020). Traditional qualitative methods of interviewing and focus groups rely on abstraction, recall and verbal reporting (Beuscher & Grando, 2009), putting pressure on people with dementia to respond in a researcher-led, question–answer format (Williams et al., 2019, 2020). The need for researchers to adapt appropriately is increasingly recognised (McKeown et al., 2010; Nygard, 2006; Williams et al., 2020; Webb et al., 2019). Co-producing research and empowering participants to be in control of the data collection process could be a solution to these challenges (Williams et al., 2020).

### Co-producing a photography project to explore post-diagnosis life with dementia

This project was co-produced between two academics from the University of Bristol and three researchers with dementia, who self-identify as the Forget Me Nots. We use the term ‘co-production’ throughout this article and the researchers with dementia are referred to as ‘co-researchers’. For us, co-production meant that all the researchers approached all aspects of the project as a team, but it was their interest and experiences of the co-researchers that were the catalyst for this research. The role of the academic researchers was to facilitate the co-researchers to conduct research that was useful and meaningful to them. This was in the service of a central tenet of co-production, the sharing of power in the research team and the development of more equitable research relationships (Bigby et al., 2014) towards what we intended to be ‘democratic, transformative and collaborative knowledge production’ (Edwards & Branelly, 2017, p. 271). When conducting co-produced research, issues relating to power sharing between academics and those with lived experience can permeate (Barton, 2005; Walmsley, 2001). We therefore had an 18-month capacity building period prior to the start of the project, to ensure the interests of the researchers with experience of dementia were at the core of the research.

The co-researchers had two key interests: (1) to explore experiences of post-diagnosis life within 2 years of diagnosis, and (2) explore how peer-support networks may help people with dementia in the period post diagnosis. Over several group meetings, the Forget Me Nots decided to use a photography workshop and focus group both as a means of collecting data and encouraging social engagement of participants. Harry Davis had found taking up photography after receiving his diagnosis to be hugely beneficial for three reasons: (1) pursuing photography gave him more impetus to leave the house, (2) the nature of medium puts the photographer ‘in the moment’ which can be grounding for someone living with dementia and (3) he gained confidence by showing himself that he could learn something new.

Our chosen method was an adapted form of ‘photovoice’, which has been demonstrated as a successful approach in enabling participants to have an active role in the research process: choosing what to take photos of and thus controlling what to share about their lives (Levin et al., 2007; Newman, 2010; Riley & Manias, 2004; Wiersma, 2011). It is important to highlight that we arrived at conducting a photovoice study organically and adapted it to our needs: the co-researchers, with no prior knowledge of ‘photovoice’, decided on the use of photography and a focus group as the best way to find out about people’s lives in a way that empowered them. Additionally, the co-researchers wanted to offer the chance for people to ‘learn photography’. This was because the co-researchers wanted to show participants they could learn new skills, to build their confidence and to offer something back for their participation.

While photovoice studies have been conducted with participants with dementia (Evans et al., 2016; Genoe & Dupuis, 2013), they have not been co-produced. Additionally, they have not explored post-diagnosis life or how photography can be a means of social engagement after diagnosis.

The aims of this project were therefore as follows: (1) to explore post-diagnosis life with dementia and (2) to use data collection methods as a form of peer support and confidence building for the participants.

## Methods

### Selecting and working with participants

A promotional poster advertising an opportunity to learn photography skills and take part in research about post-diagnosis life was circulated to local dementia organisations and key contacts to pass around their networks. We avoided recruiting from congregate settings like memory cafes or activity groups, both because they are attended by people who are already engaging in activities and because their accessibility leads to them being over-researched. Five people who fitted the inclusion criteria expressed an interest (see Table 1 for details). We made initial contact by telephone, and then met participants at their home to explain the project further. All five participants opted to take part and three elected to bring their spouses for support.

**Table 1.** Participant table.

#	Pseudonym	Type of dementia	Age	Time since diagnosis at time of project	Number of photos taken
1	Florence	Vascular	57	1 year	70
2	Jack	Frontotemporal	63	1.5 years	410
3	Lou	Vascular	77	>1 year	67
4	Miranda	Alzheimer’s	69	1.5 years	99
5	Carl	Frontotemporal	67	2 years	122

### Ethics

The project was given ethical approval by the University of Bristol’s Ethics Committee. Meeting participants before the first meeting enabled us to assess capacity. All participants in the study were able to independently give written, informed consent for research participation and for their photographs to be published. The participants retained rights and access to their photography and were given professionally printed copies of their chosen photographs, as well as digital copies of all their photographs. Participants were given £40 vouchers per session to thank them for their time.

### First workshop: photography lesson

The first meeting was held at the Royal Photography Society, where a professional photography teacher gave participants a 1 day ‘crash course’ lesson. We supplied Canon EOS 4000D cameras. The cameras could be used on an automatic mode or switched to a manual setting for participants that wanted to experiment. The photography lesson was intended to overcome potential difficulties and anxieties about using the camera. It focused on framing, storytelling, positioning a subject and lighting, with lots of examples of photography tricks and approaches to taking a photo that engages the audience.

The researchers discussed respective roles before the workshop. All researchers were to welcome participants and introduce themselves, the co-researchers would help explain the project and support the participants to feel relaxed while the academic researchers oversaw the running of the day (timing, getting lunch delivered, taxis booked, etc.). It was emphasised to the participants that the research idea and interest came from the co-researchers, and they explained about why they were interested in this project in their own words. The academic researchers were mindful to give space for the co-researchers to talk; however, the co-researchers also made it clear that they were not interested in ‘running’ the workshop but facilitating it together.

### Photography instructions

We provided easy read instructions and an annotated picture of their camera for participants to take home. The Forget Me Nots made the decision that the photography instructions should not be overly prescriptive to empower the participants to decide for themselves what kinds of photos to take. The written guidelines were:

‘*We want you to decide what to take photos of. We are interested in anything that tells us about your everyday life and how you are feeling. What makes you happy or sad? What helps or is a hindrance in your daily life? It could even be something that represents how you feel. The photos should be meaningful to you. They don’t have to works of art!’*

This guidance was given to participants alongside two examples of photographs taken for a photovoice study with people living with dementia (Evan et al., 2016): one was a literal representation of an activity and one was a metaphorical description of how the person was feeling. We also phoned participants 1 week after the meeting to provide support.

### Selecting photographs

We asked the participants to select 5–6 photographs to share with the group (Bukowski & Buetow, 2011; Mahmood et al., 2012; Novek et al., 2012). We visited all participants at home 3 weeks after the photography lesson to discuss all their photographs and finalise their choice. The initial aim was to choose photographs that most reflected post-diagnosis life, but in discussion with participants, it became clear that the meaning they attached to the photograph, or what it represented to them, was just as pertinent. Participants therefore ended up choosing the photographs they most wanted to discuss with the group.

### Second workshop: Photography discussion group

The second workshop lasted 4 hours and was held 1 month after the first meeting. Photographs were shown one at a time on a big screen, and the photographer was given an opportunity to explain why they took the photograph and the meaning behind it, with discussion and questions from the rest of the group. JW led on timekeeping and the co-research group and JD facilitated discussion between participants by asking follow-up questions (e.g. ‘we have all been there have we not?’ ‘Did you get the dog after you fell ill?’).

The last hour involved discussion of commonalities between the photographs and discussion on what the participants wanted to do with the photographs. The second workshop was video recorded and transcribed to capture the exact descriptions of the photographs, allowing for closer inspection of the participants descriptions and discussion.

### Analysis

A phenomenological approach (Van Manen, 1997) was integral to the analysis, aiming to fully reflect the meaning behind the photographs rather than their content. In this approach, ‘meaning’ is defined as ‘a result of co-creation between a researcher and researched rather than just interpretation of researcher’ (Wimpenny & Gass, 2000).

A modified, reflexive thematic analysis (Braun & Clark, 2019) took place in three stages. Firstly, the participants themselves drew comparisons between their photographs in the second workshops that formed categories (for example, *“*[my picture]*’s a bit like your picture, you know”, “well you can see the contrast with this one and the other one you took”*)*.* These comparisons were led by the participants and reflected our phenomenological approach, highlighting what the photographs stood for (e.g. nature as a comfort during difficult times) rather than surface similarities of photographic content (i.e. pictures of nature).

Secondly, the full research team met to further refine the categories the participants had identified into broader themes. JD and JW have extensive experience in qualitative data analysis, while the Forget Me Nots have experience of the analytic process from their involvement in other research projects (Williams et al., 2020; Webb et al., 2019, 2020).

Lastly, JD and JW met to scrutinise the transcripts of the final workshop alongside the themes established in stage 2. This enabled a reflective discussion where we considered our subjectivity as researchers alongside the exact words that participants used to describe their own photographs (Gough & Madill, 2012).

## Findings

### “It’s somewhat of a role reversal now”: Contrasting the past and present

Participants took photographs of their present and explained how this differed from their past. One photograph showed a hospital entrance. The participant described how he had gone from working in hospitals to attending them as a patient:

*“I’ve worked in hospitals all my life... And I just hate hospitals! Because now it’s caught up with me and I’m going every week.” (Lou)*

This role reversal from carer to cared for was a theme in these representations of how life had changed. One participant saw this is a positive aspect of his relationship with his 4-year-old granddaughter. Describing a photograph of a magnetic calendar, he explained how they bought it for her as a teaching aide, but since his diagnosis, they have started updating it together.

*“Whenever she comes to the house, she makes me go to the fridge and she watches me filling it in… She’s getting a more in-depth teaching process with things like time and that, but it’s also- it’s wonderful to see the caring coming backwards you know, from her. So, it’s sort of a cycle.”* (Jack)

**Figure 1.**
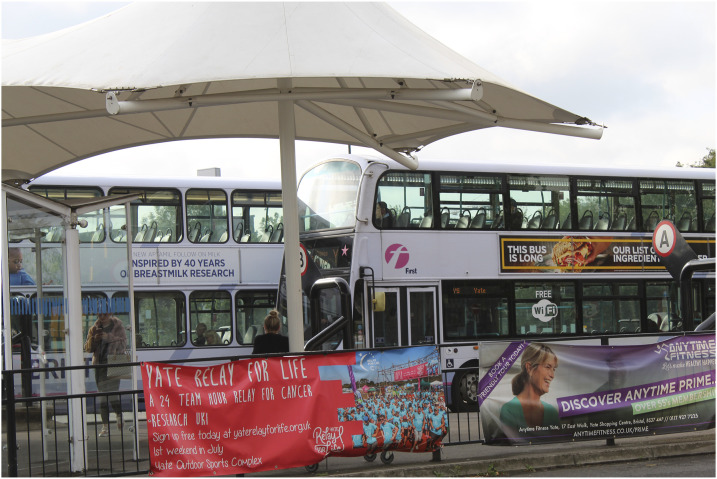
“I get off the bus and to me I'm home”.

This reversal of relationships could be more troublesome with adult children. One participant spoke about her past, having moved to a new town after her divorce, which she loved. She had moved nearer her children after her health deteriorated, but still took regular bus trips back to the town. She described her children as ‘*very, very protective’*, and the photograph of her bus (Figure 1) represented her maintaining her self-identity.

*“The kids say to me, 'where are you going’, and I say 'I'm going to [town]’... I wouldn’t want to have anybody with me, because I get off the bus and go to all the places I know and they say 'Hi [name], how are you?'. And to me I'm home.” (Miranda)*

She also described how her daughter had used the photography project to support the participant’s independence: taking photographs of all her main routes and putting them in a book with instructions. The participant liked this idea but described how it represented rapid changes to her health.

*“Now I don’t need to ring my daughter up every so often and say, I’m stood at such and such, do I go left or right? I use [the photographs], and I can go shopping on my own. Because I have lost a lot over the last 7 months, it’s going quickly.”* (Miranda)

Another participant was emotional when describing her photograph of her husband and how her strokes and resulting dementia diagnosis had impacted their relationship. She explained how he has always worked internationally, which in the past affected childcare. Now that she needs caring for, however, he is a great support.

*“I didn’t necessarily mean to capture that look, but that is how he looks. It's almost like, ‘my god, what is this woman doing?’ But there's a kindness there and he's put up with a lot, believe you me... I had my stroke, the first one, I was working, I was driving, I was all these things, and then suddenly, just like ‘bumph’. Overnight, I couldn’t drive, I couldn’t work, I couldn’t read, I couldn’t write... He makes me happy, and it wasn’t always like that.”* (Florence)

Thus, the participants used photographs to examine the juxtaposition between their past and present, and how their diagnosis had been the fulcrum on which the two are balanced and divided.

### “There’s a gauze in front of your memories”: Representations of dementia thought processes

Imagery was employed to illustrate the way dementia affected participants’ cognitive functioning. One participant described his photograph depicting a flower viewed through a rain-streaked windowpane.

*“I was trying to focus on the flowers… and I thought, 'well I can’t quite see it', which is a little bit like when the dementia sort of descends on you, there's almost like a gauze that comes in front of your memories... And you have to concentrate a little bit more on everything that you hear and see.*” (Jack)

Another participant took a similar approach, taking a photograph of a small flower that was in sharp focus, while the rest of the foliage was blurred. He used the visual concepts of ‘in focus’ and ‘out of focus’ to represent his cognitive processes. In an exchange with another participant, he explained:

*Carl: You know, I get things out of focus*

*Jack: You can concentrate okay on one bit*

*Carl: Yeah, but everything else…*

**Figure 2.**
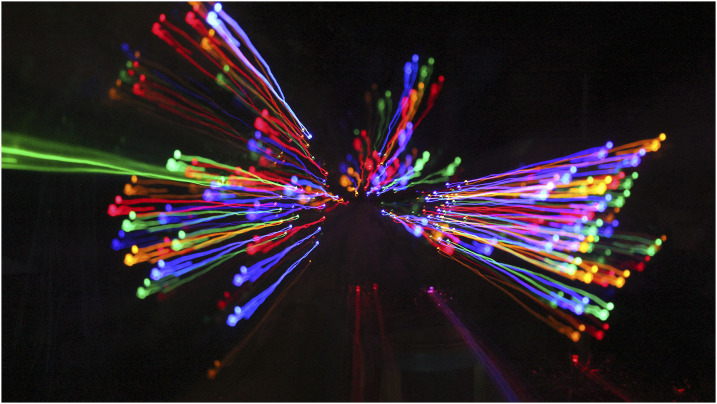
“It becomes more blurry”.

One participant explained his photograph of brightly coloured lights on a black background that became gradually blurrier (Figure 2).

*“The dots that you’ve got in the back there, that’s the initial memory. So that’s your childhood if you like, where it’s all quite clear. And as you’re getting through your life it becomes more blurry... So, my memory can remember things that happened when I was a child, when I was younger, specific events, in my past I can remember like it’s only happened yesterday. But for instance, getting here to this meeting I can’t remember.”* (Jack)

The same participant also took a picture of what appeared to be giant pillars, flanked by shadows. On closer inspection, a loose screw is visible in the foreground revealing the ‘pillars’ to be in fact stools.

*“Until you see the screw you don’t actually know how big the stuff is. It's a little bit like having dementia… you see something but you don’t always put it into perspective in your life you know and it's not until you see a tiny bit of detail or what have you that the picture comes a bit clearer for you.”* (Jack)

Participants therefore used the composition and subject of their photographs as metaphors of how dementia caused them to see the world differently, making opaque and distant what was once clear.

### “It’s just such a wonderful medicine”: Facilitators of support and joy

Participants contributed photographs that illustrated parts of their lives that lifted them out of negative responses to their diagnosis. One participant photographed a fairy garden she made for her granddaughter. Being involved in a creative activity that was intended to being happiness to a loved one was identified as a positive process.

*“Because she knows that Nanna’s not well, and of course my son says ‘oh Mum’s away with the fairies’, which didn’t help! But it did in the context of this… I enjoy doing stuff like that, a lot of craft stuff. And that made me feel better.”* (Miranda)

Grandchildren were identified as an important source of positivity because they did not treat participants differently since their diagnosis, which stood in contrast to their adult children.

*“She doesn’t really treat me like Nanny, because we’ve always done stuff together… She comes in, does her thing. And there’s no, what you doing, or can I do this? Whereas my daughter is gorgeous, she’s so good – but she’s so bossy!”* (Miranda)

Grandchildren could also alter low moods brought about by negative feelings associated with dementia.

*“When they come in through the door you know, you get down on your knees and they jump at you... And they’re so excited like, and all your darkness disappears.”* (Jack)

Three participants had purchased dogs after their diagnosis and described their positive impact. As one participant’s spouse observed about her husband:

*“Even when you’re down, [the dog] ends up making him laugh”* (Lou’s wife)

Pets helped the participants retain a sense of belonging and usefulness to others, as well as providing a source of joy in darker times. The responsibility of being a pet owner also provided the participants with opportunities to be independent and active.

*“Particularly when I had my first stroke, everyone does everything for you… But I think people can sort of kill you with kindness sometimes… And* [dog name] *demands- well doesn’t demand it, but you know that he needs a walk, and when I have to do something, I can do it.”* (Florence)

**Figure 3.**
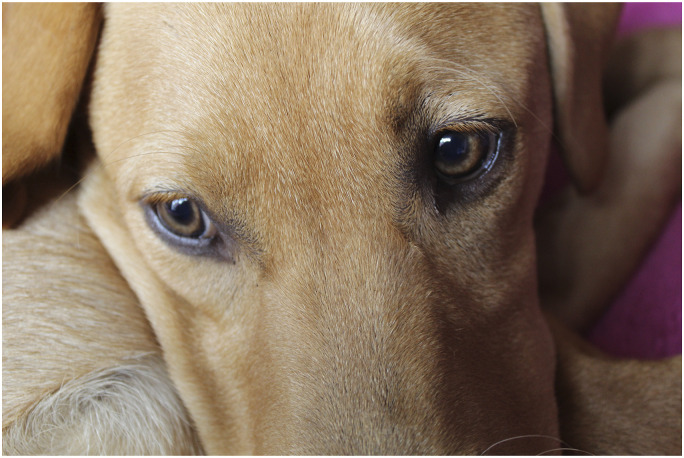
“You have a reason for being”.

*“You’re also given a purpose again, if you like, in that you have a reason for being. And you get up and feed them and let them out.”* (Carl, figure 3)

Thus, photographs of pets and grandchildren enabled participants to reflect on the responsibility inherent in these relationships, which created reciprocal, positive, emotional support.

### “It’s as easy as breathing to go there”: Emotional states associated with dementia

The photographs also reflected dark moments. One participant took a photograph of raindrops and reflected on how the weather mirrored his mood that day.

*“I was pretty depressed one day and I took this* [photograph] *which is a really heavy rain storm… to me, it sort of went to how I was actually feeling about my condition and all that sort of stuff”* (Carl).

One participant described an affinity he felt with a snail on the ground, framed by an expanse of concrete.

*“You’ve got to sit and think about everything and try and put it into the right context. And that’s how I feel sometimes. I feel like a little lost snail there, hasn’t got a clue where he’s going, what he’s doing… [the snail] will get there in the end, we just don’t know when the end is going to be”.* (Lou)

Uncertainty about the future was a common theme. This participant explains his photograph of a forest path among the trees:

*“The whole point of this was, where we are at the moment – we’re on the grass. We’re here and now, I can see where I’m at…The way forward is some trepidation, but also maybe something magical, like the lion, the witch and the wardrobe at the end of it. Nobody knows, I have no idea where that path is going to take me.”* (Carl)

Other participants hoped that the future will be more positive than their present. As the wife of one of the participants read out a prearranged description of a photograph he had taken of a full moon in the darkness.

*“He said that's how I felt when they told me* [my diagnosis]*. Everything was black and you were trying to get to the light in the end”.* (Lou)

**Figure 4.**
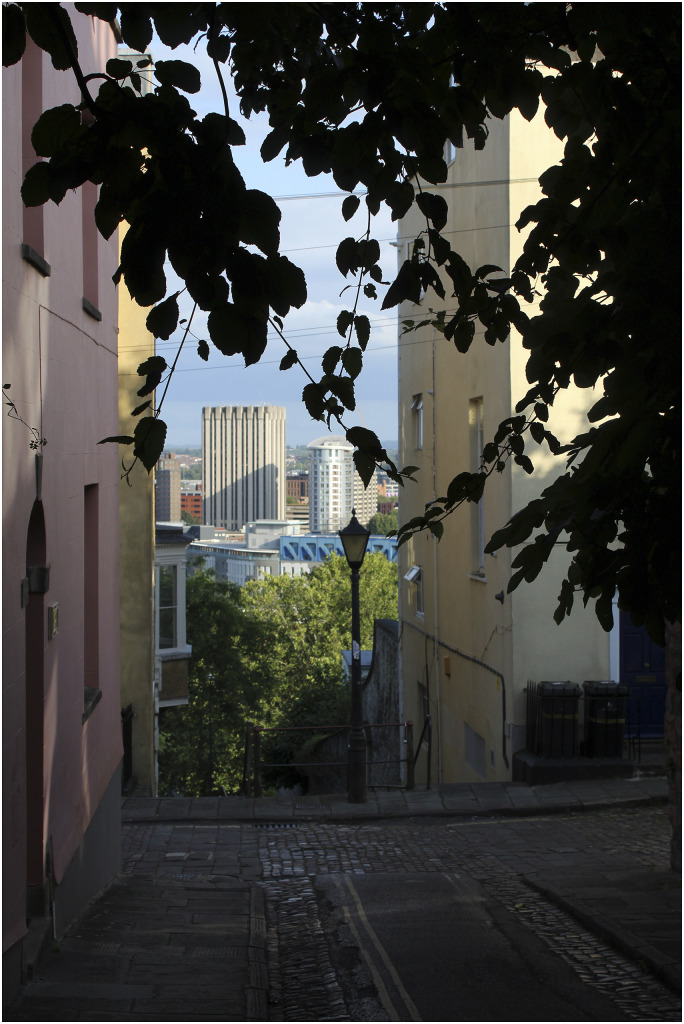
“I know there’s light out there”.

Another participant’s photograph was framed by dark shadowy branches, looking out into a brightly lit cityscape in the distance (Figure 4).

*“I’m standing on the hill, and I’m surrounded by these dark branches… But I know there’s light out there, that there’s hope somewhere, no matter how black you might be feeling… It’s very hard when you’re encased in feeling depressed or low, I would say. It’s very hard to see beyond that”.* (Florence)

One photograph took a starker look at moments of depression: a self-portrait of the participant hunched over in a depressed posture. The effect was exaggerated by the black and white colour palate. He explained:

“*It reflects a point that probably we’ve all faced at one point after our diagnosis… It’s all very grey, grainy, there’s no colour anywhere…and depression is, it’s something that potentially all of us can inhale… But it’s such a hard thing to overcome and get rid of, and exhale depression. And so it lingers.*” (Jack)

These photographs encapsulated the mixture of complicated and uncertain feelings, hope and depression, light and dark, which were precipitated by the diagnosis.

### “A rose is always a rose”: taking pleasure and reassurance from the outside world

Participants reflected on the joy in the outside world, from nature’s reoccurring patterns, indifferent to their diagnoses. When talking about their photograph of a robin on a fence, one participant described the robin’s appearance each year had taken on particular meaning since her neighbour died a year ago.

*“We were such close friends, and she used to come round, and I'd bring her round cos she couldn’t walk, and we used to sit together. But [the robin] still comes… I just sit and chat to him”.* (Miranda)

**Figure 5.**
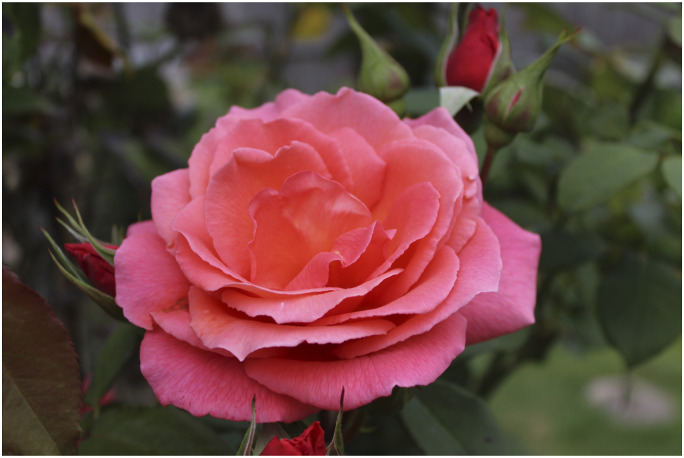
“A rose is always a rose”.

One participant took a close-up picture of a rose (Figure 5). The detail of the picture reflected his reverence for nature and its reassuring permanence.

*“It's the sheer beauty of it. You don’t really realise it, but it's always there…It never changes. If you go out and see a rose, it's always a rose… it’s stability”* (Lou)

The sense of the permanence of the outside world was also present for one participant in their photograph of a cat-like metal face carved moulded into the arm of a park bench.

*“I like the shape of the head, the way it curves… and I like the idea that something as simple as park bench you can get pleasure from... it doesn’t have to be a dramatic building or a huge object, it can be something quite simple...solid. Something to hold on to”.* (Florence)

The participants thus joy and stability in objects in the outside world, specifically in relation, and possibly in reaction, to receiving a diagnosis of dementia.

## Discussion

Our exploration of the everyday experiences of people recently diagnosed with dementia using photography provided some insight into how participants were experiencing and adapting to a period of great change. The photographs reflected the differences between their past and present with dementia, how dementia affects their thought processes, the facilitators of joy in their lives, their darker moments and uncertainty about their future and the reassuring role of nature.

### Experiences of living with a recent dementia diagnosis

The findings support and contribute to our current understandings of how the early stages of dementia are experienced. Receiving a diagnosis is typically related to a period of upheaval and change (Bunn et al., 2012; Aminzadeh et al., 2007; Xanthopoulou & McCabe, 2019; Clare et al., 2008). The participants appeared to be undergoing what Keady and Gilliard (1999) refer to as an active process of learning to live with dementia. Previous research has noted how people living with dementia often find resourceful ways to cope (Harman & Clare, 2006; Sorensen et al., 2008), which for our participants involved drawing on important relationships, such as with pets and grandchildren, and taking solace in the reliability of the natural world. Participants also expressed hope through looking to a possibility of a brighter future, demonstrating a positive discourse around living with dementia that is often missing in public representations (Mitchell et al., 2013). However, this hope was tinged with uncertainty at what was to come. A dementia diagnosis can be experienced as ‘a death sentence’ (Parsons-Suhl et al., 2008, p. 40). While this was not the case with the participants, they were all nevertheless severely affected by their diagnosis, which had resulted in experiences of depression and low moods. Participants frequently drew upon their past to make sense of their present, describing how who they were contrasted with who they are. This grappling with changing roles, lives and identities brought to the fore the difficulty of adapting to post-diagnosis life.

### Photography as a research method

An important finding of this study was how the participants with dementia were able to use photography to create metaphors that represented their experiences. For example, Miranda valued her bus trips to her old town because it represented her link with her past life. Her photograph of the bus was therefore a totemic symbol of freedom. Likewise, other participants shared images which were metaphors for their emotional states, hopes, fears and anxieties. Hence, their photographs facilitated deeper knowledge of their lives by capturing insights that may not otherwise have been revealed (Palibroda et al., 2009). This was because they were able to choose what they wanted to share (Levin et al., 2007; Newman, 2010), and the use of creative photography enabled them to communicate in a visceral way. The success of photography as a method echoes previous research that found photovoice provides an opportunity for people to create a record and to engage in reflection (Wang & Burris, 1997; Wiersma, 2011, p.206), as well as other research that has used the arts to ‘reclaim citizenship’ in dementia, citing artistic activities as key to allowing people with dementia tell their stories in their own terms (Dupuis et al., 2016).

### Photography as a stimulus for social engagement

Post-diagnosis peer support can reduce isolation and support self-worth by enabling people to contribute to helping others through their post-diagnosis period (Kelly & Innes, 2016; Clare, 2002). However, none of the participants shared photographs of support groups or memory cafes or saw them as having a role in their diagnosis journey. People living with dementia greatly value opportunities for social and recreational activities (Chester et al., 2018), but attending dementia-focused congregate groups can be unappealing for people with a recent diagnosis, who may fear having little in common with other participants except memory problems (Mason et al., 2005; Brodaty et al., 2005).

Hence, the major addition of this study to the existing literature was that the photography was chosen not only as a method of data collection but as a stimulus for leaving the house, learning new skills and sharing experiences with peers. Our project demonstrates that combining research activities with social activity, where participants can engage with something new, can be successful. This draws on previous research showing that photography can enable participants to express emotions while also building confidence (Foster-Fishman et al., 2005; Radley & Taylor, 2003). Photovoice studies often use disposable cameras (e.g. Genoe & Dupuis, 2013), citing the simplicity of the camera design making it easier to take part. However, we provided the participants with a DSLR camera, reasoning that the project was designed to offer participants the chance to gain new skills and that the opportunity to learn something new may motivate people to take part, who otherwise may not be interested in participating. This was confirmed by participants to be a hugely beneficial outcome of this project. The number of photographs taken (Table 1) shows the enthusiasm the participants had for the method. Several participants bought their own cameras, with one joining a photography club. Florence decided to join a dance group for people living with dementia: a decision which she explicitly tied to having had a positive confidence-building experience in our research. Jack has subsequently set up his own website supporting people with dementia to navigate post-diagnosis life, through which he has also shared his photographs taken in the project [https://minusmymemory.com/]. Speaking about the effect of involvement in the research, Lou’s wife remarked:

*“Since he’s been here he’s changed… He wants to go to the memory club, he wouldn’t go before. We’ve got a camera, he’s using it. Next week he’s joining a photography class…Before we couldn’t find anything that would interest him. Last time [in the photography lesson] he didn’t say a word … He said, ‘you have to speak for me, I can’t speak’. He’s spoken today [in the second workshop]. That’s what this has done…you’ve opened something up with him.”*

This quote from Lou’s wife also highlights another barrier to post-diagnosis social engagement: many people with dementia do not have the confidence to attend peer-support groups. Three participants brought their spouses as social support and these happened to be the three male participants. Men have been shown to be harder hit by loneliness in older age (Willis et al., 2019) and this is likely to be worse when men are diagnosed with dementia, as people’s hobbies and interests are often compromised post diagnosis (Muò et al., 2005). Creating post-diagnosis support groups that focus on learning new skills may therefore work particularly well in engaging men after receiving a dementia diagnosis.

### The role of co-production

The success of the project design in encouraging social engagement stemmed entirely from the role of the co-researchers with dementia. In traditional research production, disabled people are often positioned as passive objects (Oliver 1992). In contrast, working with our co-researchers led organically to a more emancipatory research approach in which the ‘objects of research’ themselves actively define the research agenda and control the outcomes (Barnes, 2003; Barton, 2005). For the co-researchers, the method of data collection had to be an instrument of change in and of itself and should level any power imbalances between a researcher and researched. This lead not only to the choice of photography as a method, but also to the use of a professional photographer to run the photography lessons, which ensured a high-quality learning experience for all involved. The co-researchers were key in encouraging reflection around the photographs in the second focus group, drawing on their own experience with dementia to empathise with the participants and allow for more in-depth discussion.

### Reflections and future research

While the co-production approach made for extremely successful outputs to this project, it is important to reflect on the challenges. The key barriers to truly co-produced research are time and resources, both are integral to forming open, trusting working relationships. The academic researchers and co-researchers living with dementia had the luxury of working together for years prior to this project, which is something not all research institutions have built into their organisational structure. It is important that research projects focused on designing care interventions for people with dementia include those living with dementia from their inception, but it is necessary to build the time and resources in to make this happen.

Our current project demonstrates the challenges that people with dementia experience post diagnosis. It shows that not only is peer support very important for people with dementia in this period, but that learning new skills is not only possible but hugely beneficial. Future research could explore the factors that affect this positive outcome more systematically.

As the impetus for the research came from the co-researchers’ experiences and insights into this transitional period, we conclude with their insights on the project.

## Postscript by the Forget Me Nots

Photography gave the participants the opportunity to branch out, to get on with their lives. It helped them understand more about each other that they were different but still the same in some ways. When I took up photography, I felt it needed to get out of the house. Photography gives you that reason to go out and do something. When you are diagnosed with dementia, you are told what you are *not* capable of doing. The key thing is that there are things you can do, not endless things you cannot. The participants for this project were sat at home because they were not told they could do something. It’s not until you are told you can do something that you think you can do it. When I took up photography, I felt like I could do something. You can learn new skills – there is a part of your brain that is great. You can learn new things, and that is what we wanted to show people.

I think these photographs brought out their emotions. The photographs gave them the opportunity to speak about things. It made them feel better because they got something out. They opened up because we were chairing the session – we all had something in common. And I think we all wanted to learn from each other. If you take a photograph, and someone says straight away, ‘why did you take that?’, you do not know! But they had a chance to think about what why they took these photos. They were not put on the spot. I do not think we would have got the same information from them without the photos they took. A picture speaks a thousand words. It’s their lives, what they live for. That’s the thing about dementia – once you are over the initial shock of the diagnosis, your brain does work in a different way. You get to see things in a different way. A photograph is a way of recording that difference.
